# Impact of Antiplatelet Therapies on Patients Outcome in Osteosynthetic Surgery of Proximal Femoral Fractures

**DOI:** 10.3390/jcm8122176

**Published:** 2019-12-09

**Authors:** Michael Humenberger, Matthias Stockinger, Stephan Kettner, Jolanta Siller-Matula, Stefan Hajdu

**Affiliations:** 1 Department of Trauma Surgery, Medical University of Vienna, Vienna 1090, Austria; Michael.humenberger@meduniwien.ac.at; 2 Department of Trauma Surgery, Tauernklinikum Zell am See, Zell am See 5700, Austria; matthias.stockinger.dr@gmail.com; 3 Department of Anaesthesia, Intensive Care Medicine and Pain Medicine, Krankenhaus Hietzing, Vienna 1130, Austria; stephan.kettner@meduniwien.ac.at; 4 Department of Cardiology, Medical University of Vienna, Vienna 1090, Austria; jolanta.siller-matula@meduniwien.ac.at

**Keywords:** proximal femoral fractures, antiplatelet therapy, hip fractures, osteosynthesis, clopidogrel, dual antiplatelet therapy

## Abstract

Background: Proximal femoral fractures should be treated in a timely manner. Affected patients often require antiplatelet therapy (APT) due to cardiovascular diseases. Guidelines recommend 5-7 days APT interruption for elective surgery. Early osteosynthetic surgery of proximal femoral fractures despite of APT should be considered. Aim of the study: To evaluate whether early osteosynthetic surgery despite of APT is associated with increased blood loss, complications and mortality. Methods: Data of patients with proximal femoral fractures, who were treated by osteosynthesis at the Department of Trauma Surgery at the Medical University of Vienna were collected retrospectively. Study groups were formed by time to surgery and APT interruption. The primary endpoint of the study was the perioperative blood loss. Secondary endpoints were complications, 30-day and 1-year mortality, time to surgery, and the total length of hospital stay. Results: The osteosynthetic treatment of proximal femoral fractures despite of APT resulted in a shorter time to surgery (13.8 vs. 66.0 h; p < 0.01). In patients on APT, the TBL (total perioperative blood loss) was higher without need for revision or an increase in the need for packed red blood cells if surgery was performed within 24 h after admission. APT had no significant influence on mortality. Patients who underwent surgery within 24 h after admission had a lower mortality. The complication rate was higher in patients who underwent surgery later than 24 h after admission. Conclusions: Surgery within 24 h after admission, regardless of APT, resulted in a shorter hospitalization length and was associated with less common complications and a lower mortality.

## 1. Introduction

Proximal femoral fractures represent one of the most common injuries among the elderly population [1]. The 30-day mortality rate following proximal femoral fractures is 1.7–6.9% [2] and 14–36% within the first year [3,4]. Comorbidities have the greatest influence on the postoperative mortality rate [5]. Affected patients are often multimorbid and require antiplatelet therapy (APT) due to coronary diseases, peripheral arterial diseases or ischemic strokes. Patients who receive percutaneous coronary interventions (PCI) with stent-implantation require a dual APT with acetylsalicylic acid (ASS) plus clopidogrel or a new platelet P2Y12 receptor antagonist, like prasugrel or ticagrelor. The European Society of Cardiology (ESC) suggests a dual APT for 4–6 weeks after implantation of bare-metal-stents and 12 months of dual APT after implantation of drug-eluting stents. Patients who sustain proximal femoral fractures after PCI and stent-implantation pose a special problem for the surgical team. On the one hand, the interruption of APT increases the risk for life-threatening stent-thrombosis, yet on the other hand continuation of APT is associated with an increased risk for perioperative blood loss and an increased need of blood transfusions [6,7]. Therefore, guidelines recommend a 5–7-day interruption of APT prior to elective interventions to reduce the risk of blood loss [8,9]. The ESC suggests an individual risk evaluation by comparing the perioperative bleeding risk and the risk for thromboembolic events [10]. The ideal timing for surgery depends on the indication for APT, on the type of the surgery and on the general condition of the patient [11]. However, in patients who suffered a proximal femoral fracture, the long preoperative period leads to immobilization and increases the risk of systematic complications including thromboembolic events, pneumonia and urinary tract infections, which in turn increases morbidity and mortality rates [12]. Proximal femoral fractures are therefore considered as semi-urgent surgeries and surgery should be performed within 48 h after the fracture occurs [13]. In addition, a prolonged time to surgery implies extra mental stress for the patients. For these reasons, early surgery of proximal femoral fractures despite of APT should be considered. Due to a lack of prospective data, we performed a retrospective analysis of patients on APT who underwent early surgery by osteosynthesis for proximal femoral fractures. 

## 2. Methods

This retrospective cohort study includes data from patients who suffered proximal femoral fractures and were treated by osteosynthesis between January 2013 and December 2015 at the Department of Trauma Surgery of the Medical University of Vienna. The hospital internal databases were used to collect data to enable the statistical evaluation with Excel 2016 and SPSS 23. Osteosynthetic surgery was performed as soon as possible. Patients on APT were either prepared for surgery the next days or early surgery was performed despite of the APT. If surgery was not performed early, the APT was interrupted on case-by-case basis. In patients at high ischemic risk or recent coronary interventions, APT was not interrupted. If patients received APT for primary prevention of thrombotic events, APT was interrupted prior to surgery and was restarted after secondary bleeding was ruled out. The attending orthopedic trauma surgeon and the attending anesthesiologist made the decision of early or delayed surgery. Age, sex, date of admission, time to surgery, total length of stay, ASA grade, type of surgery, comorbidities, systemic and surgery associated complications, mortality, pre- and postoperative hemoglobin levels, as well as blood transfusions, were evaluated. To estimate the total blood loss, the hemoglobin balance formula was applied by including the total blood volume of the human body, the hemoglobin difference and the hemoglobin content of blood transfusions [14]:

Hemoglobin balance: Hb_totalloss _= Blood volume (mL) × (Hb _before surgery_ − Hb_ after surgery_) × 0.001 + the total amount of hemoglobin of transfusions (52g Hb per blood transfusion)

[14]

The study contains two groups of consecutive patients: the study group, including patients on APT and the control group, in which patients had not obtained APT. The study group was divided into subgroups based on the type of APT: aspirin, clopidogrel or dual APT. These subgroups were further subdivided in regard to whether the APT was interrupted for more than 24 h prior to surgery. 

### Statistics

Sample size and power: Considering a 13% absolute difference in mortality between patients who underwent surgery within 24 h vs. those who underwent surgery after 24 h, we calculated a sample size of at least 87 per group with a sampling ratio of 5/1 to achieve a power of 80% with a two-sided alpha of <0.05. Groups were compared by using the binominal test, Chi^2^-test, t-test or Mann-Whitney test as appropriate. Multivariate Cox-regression analysis was used to adjust for group differences. Adjustment was performed for following variables: sex, ASA score, type of surgery, age, APT, APT interruption and time to surgery. A Chi-squared Automatic Interaction Detection (CHAID) analysis was performed to identifiy predictors for high blood loss. Kaplan-Meier curves were used for time- to event data. *p*-values less than 0.05 were considered as statistically significant. 

## 3. Results

We included 396 patients with a mean age of 80.4 years. Fifty six percent of all patients were treated by APT. Patients on APT were on average seven years older than patients without APT. Screw fixation was performed in 29 patients (7.3%), 53 (13.4%) were treated by dynamic hip screw (DHS) and 314 (79.3%) by intramedullary nailing. Surgical time was 57 minutes (mean 57, min. 18, max. 180, SD 25) and was not influenced by APT or time to surgery after the fracture (*p* > 0.05). Only patients with isolated fractures due to a low-energy trauma like a fall were included. One hundred and eighty nine patients took ASS, 20 clopidogrel, 12 dual APT and 174 had no APT. (Table 1 and Table 2).

**Figure 1:**
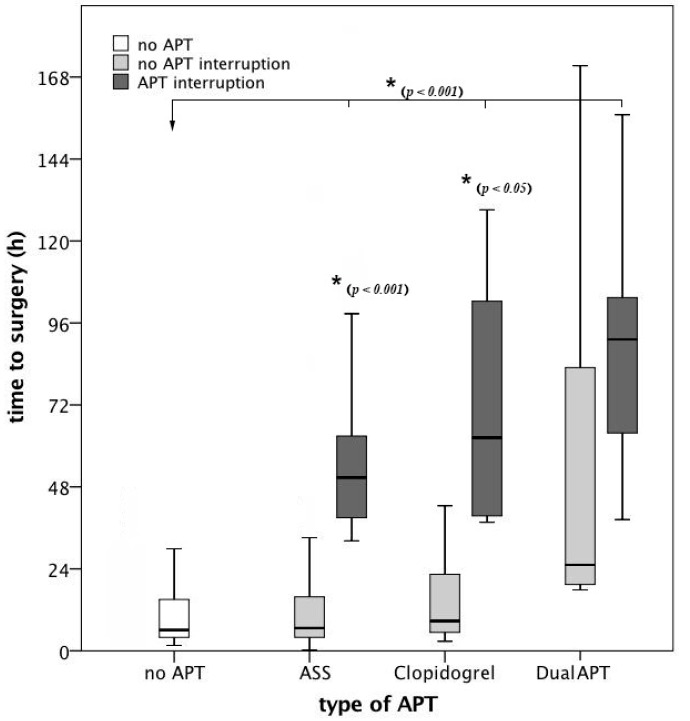
Time to surgery broken down on the type of antiplatelet therapy (APT) and APT interruption. After interruption of the APT, the time to surgery was significantly longer compared to patients without APT and compared to patients without interruption of the APT. Compared to patients without APT, there was no significant difference in time to surgery in patients on APT without interruption of the APT. ASS: acetylsalicylic acid.

In total, 308 (77.8%) patients with proximal femoral fractures were operated within 24 h after admission, 69 (17.4%) within 72 h and 19 (4.8%) waited longer than 72 h for surgery. Patients on APT had a significantly longer time to surgery than patients without APT (mean 21.8 vs. 14.8 h; *p* < 0.05). If the APT was interrupted, the time to surgery was significantly prolonged compared to patients without APT and to patients on APT without interruption of the APT. If the APT was not interrupted, there was no significant difference in time to surgery compared to patients without APT (Figure 1). 

**Figure 2:**
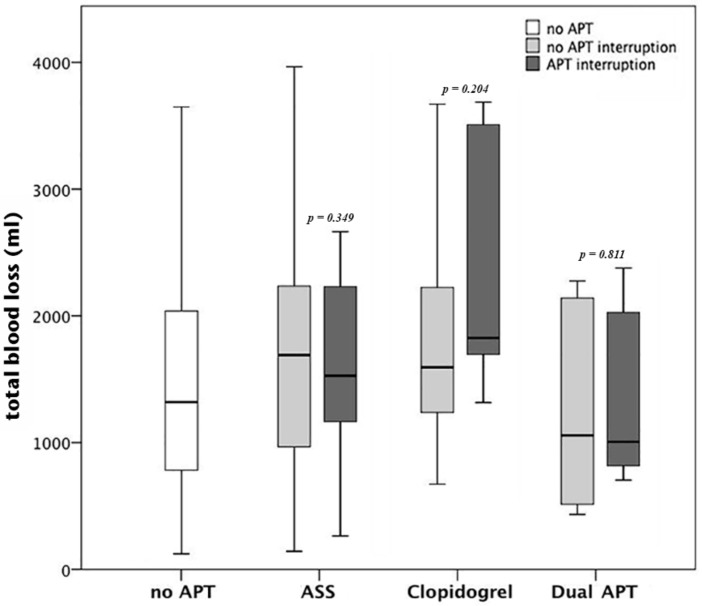
Total blood loss (TBL) broken down to the type of APT: there was no difference in TBL between patients without APT and patients on acetylsalicylic acid (ASS), clopidogrel or dual APT, regardless of APT interruption (*p* < 0.05 for each comparison).

The total blood loss (TBL) was significantly higher in patients, who were treated by intramedullary nailing, compared to patients, who were treated by extramedullary methods like screw fixation or DHS (1758mL, 179–7893 vs. 1124mL, 122–6089, *p* < 0.01). However, there was no difference in TBL between patients without APT and patients on ASS, clopidogrel or dual APT, regardless of APT interruption (*p* < 0.05) (Figure 2).

**Figure 3:**
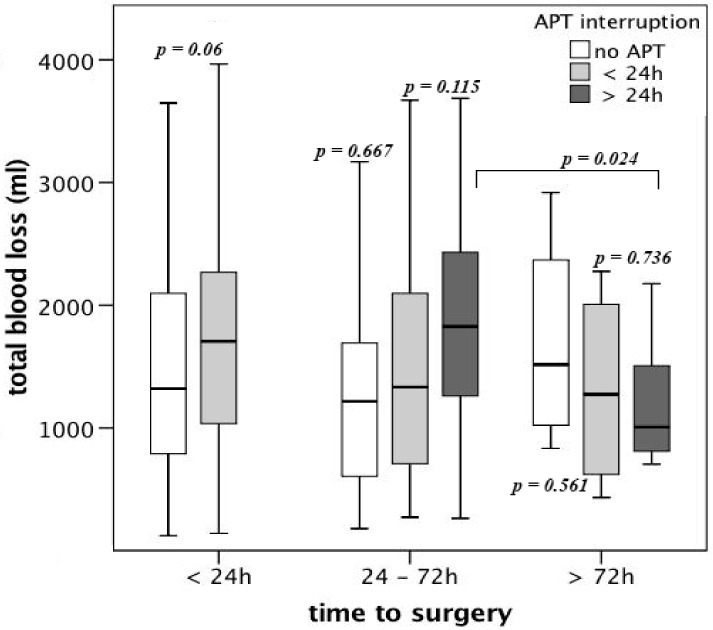
Total blood loss (TBL) in patients on APT broken down to the time to surgery after admission and APT interruption

There was no difference in total blood loss between different time points in patients without APT and in patients with ongoing APT (*p* > 0.05, Figure 3). Compared to patients without APT, patients on APT had a higher TBL if surgery was performed in less than 24 h after admission (1493 mL, 122–3648 vs. 1780 mL, 142–6089; *p* < 0.05). Patients with APT interruption, who underwent surgery in less than 72 h after admission had a significantly higher TBL compared to patients who underwent surgery after more than 72 h (1869 mL, 263–3687 vs. 1195 mL, 704–2176; *p* < 0.05, Figure 3).

**Figure 4:**
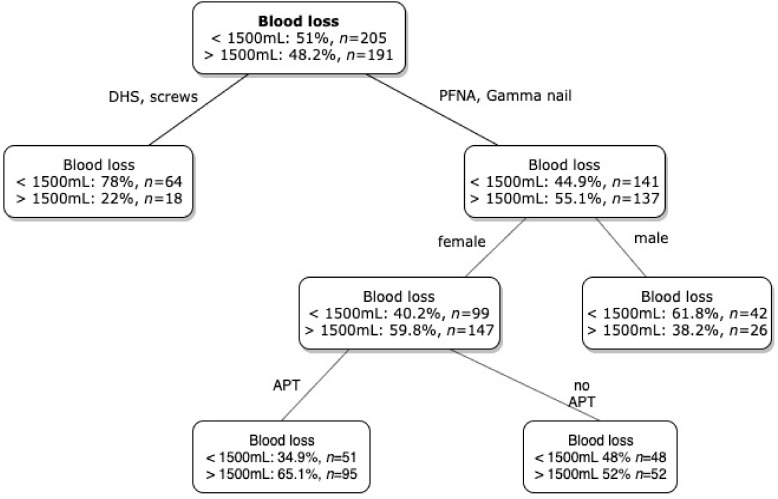
Chi-squared Automatic Interaction Detection (CHAID) analysis for discriminators of blood loss over 1500mL. DHS: dynamic hip screw, PFNA: proximal femoral nail antirotation, APT: antiplatelet therapy. Intramedullary surgery, female gender and APT were predictors for high blood loss.

By applying a logistic regression model, including sex, ASA score, type of surgery, age decades, APT, APT interruption and time to surgery, we identified female gender (*p* = 0.001, OR 4.023, 95% CI 1.824, 8.871), intramedullary nailing (*p* = 0.001, OR 3.768, 95% CI 1.721, 8.249) and age decades (*p* = 0.004, OR 1.618, 95% CI 1.170, 2.237) as predictors for high blood loss (>1500 mL TBL). ASA score, APT and APT interruption were no predictors for high blood loss. By performing a Chi-squared Automatic Interaction Detection (CHAID) analysis, we identified the type of surgery as predictor for high blood loss (>1500 mL), followed by female gender and APT (Figure 4). 

Patients on APT, who underwent surgery within 24 h had no increased need for packed red blood cells (RBC) compared to patients on APT who underwent surgery within 72 h or later (1.6 vs. 1.6 vs. 1.4, *p* > 0.05). There was no difference in the total number of RBCs between patients without APT and patients receiving ASS (1.15 vs. 1.5, *p* > 0.05). Patients on clopidogrel had a significantly higher need for RBCs, compared to patients without APT (2.15 vs. 1.5, *p* < 0.05). Patients on dual APT had a higher need for RBCs compared to the control group, without reaching statistical significance (1.67 vs. 1.15, *p* > 0.05). There was no case of major bleeding, needing revision surgery or death. 

Urinary tract infections (UTI) and pneumonia were recorded in 97 patients, whereby 86 (21.7%) sustained UTI, 11 (2.8%) pneumonia and six (1.5%) suffered from both. There was no difference in the incidence of UTI or pneumonia between patients on APT and patients without APT. In patients with interrupted APT, the incidence of UTI and pneumonia was higher compared to patients with ongoing APT (UTI: 28.1% vs. 20.1%; pneumonia: 6.3% vs. 2.6%). Patients on APT who underwent surgery within 24 h suffered less UTI than patients who underwent surgery later than 24 h (18% vs. 30%) after admission. Minor surgical site complications, like prolonged secretion of the wound, were found in 16.9% of all patients. There was no difference between patients on APT and patients without APT. In patients on APT, less surgical site complications were found if APT was continued compared to APT interruption (16.4% vs. 25%). Regardless of APT, patients who underwent surgery later than 24 h after admission suffered more surgical site complications compared to patients who underwent surgery within 24 h after admission (16.1% vs. 21.7% in patients on APT and 15.6% vs. 21.6% in patients without APT). The overall complication rate of surgical site and systemic complications was higher in patients who underwent surgery later than 24 h after admission (50% vs. 38.5%).

Six patients suffered a thromboembolic complication. Three on APT (1.4%) and three without APT (1.7%). In the study group, two patients with thromboembolic complications underwent surgery within 24 h and one within 72 h. One patient, who was treated by ASS due to a coronary heart disease and underwent surgery within 24 h after admission deceased due to a myocardial infarction three days after surgery. One patient, also treated by ASS, who underwent surgery within 4 h after admission, died due to pulmonary embolism three days after surgery. Another patient suffered a non-ST-elevation myocardial infarction prior to surgery. She underwent surgery 51 h after admission without further complications. 

**Figure 5:**
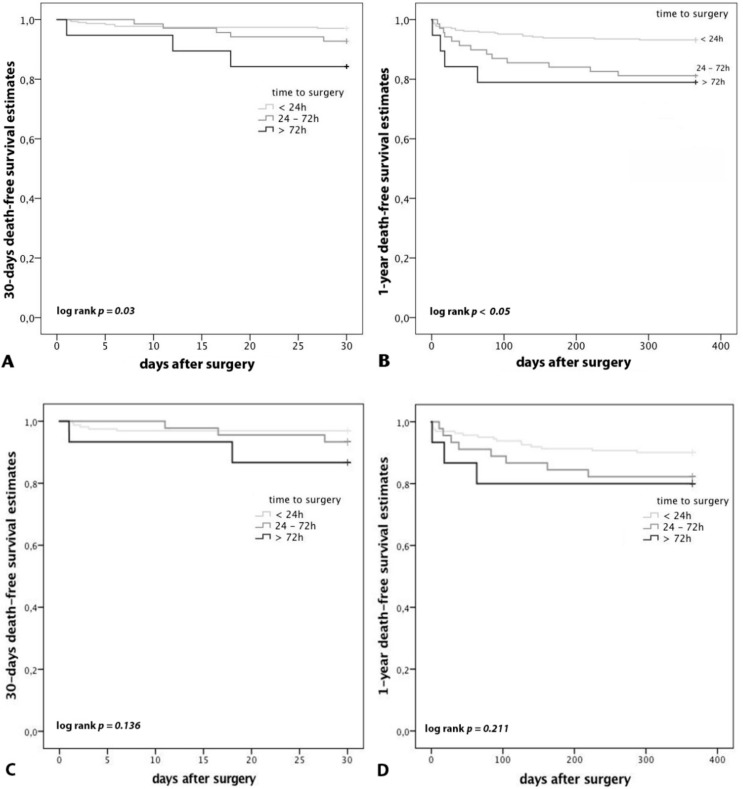
Mortality subdivided by the time to surgery after admission and by APT. (**A**): 30-day mortality of all patients; (**B**): 1-year mortality of all patients: Patients who underwent surgery within 24 h had a lower 30-day (2.9%) and 1-year (6.8%) mortality rate, compared to patients who underwent surgery between 24 and 72 h (30d: 7.2%, *p* > 0.05, 1y: 18.8%, *p* < 0.05) and patients who underwent surgery later than 72 h after admission (30d: 15.8%, *p* < 0.05, 1-year: 21.1%, *p* < 0.05). (**C**): 30-day mortality of patients on APT; (**D**): 1-year mortality of patients on APT: In patients on APT, the time to surgery showed a similar pattern of higher survival if surgery was performed earlier without reaching statistical significance.

The 30-day mortality was 4.3% in total. Male gender (*p* = 0.01, female: OR 0.247, 95% CI 0.086, 0.712), age (*p* = 0.036, OR 1.062, 95% CI 1.004, 1.123), and time to surgery (*p* = 0.028, OR 2.290, 95% CI 1.093, 4.797) were identified as predictors for an increased 30-day mortality by performing a logistic regression analysis including sex, ASA score, type of surgery, age decades, time to surgery and APT as independent variables. There was no difference in the 30-day mortality rate between patients on APT and patients without APT (4.5% vs. 6%; *p* > 0.05). Compared to patients without APT, patients on APT had a higher 1-year mortality without reaching statistical significance (12.2% vs. 6.3%; *p* = 0.051). In patients on APT, APT interruption did not lead to a difference of the 30-day or the 1-year mortality. The time to surgery had a significant effect on mortality in all patients. Patients who underwent surgery within 24 h had a lower 30-day (2.9%) and 1-year (6.8%) mortality rate, compared to patients who underwent surgery between 24 and 72 h (30d: 7.2%, *p* > 0.05, 1y: 18.8%, *p* < 0.05) and patients who underwent surgery later than 72 h after admission (30d: 15.8%, *p* < 0.05, 1-year: 21.1%, *p* < 0.05). (Figure 5A,B). In patients on APT, the time to surgery showed a similar pattern of higher survival if surgery was performed earlier without reaching statistical significance (Figure 5C,D). Patients who underwent surgery later than 72 h after admission had a significantly longer length of hospital stay compared to patients who underwent surgery earlier (14.9 days vs. 21.9 days; *p* < 0.001).

## 4. Discussion

Our study demonstrates that early osteosynthetic surgery of proximal femoral fractures in patients on APT results in a shorter time to surgery, in a shorter total length of hospital stay, no increased need for RBCs, no increased incidence of bleeding complications, in a lower 30-day and 1-year mortality and in less common complications compared to delayed surgery, regardless of APT. 

The ESC-Guidelines of 2017 recommend a dual APT for at least one month, independently of the type of implanted stent [15]. If surgery cannot be delayed, surgery should be performed in hospitals where catheterization laboratories are available 24/7 to treat perioperative thrombotic events. In patients at high ischemic risk, dual APT is recommended for at least six months. An interruption of the dual APT within this period leads to an increased risk of severe cardiac complications, such as stent thrombosis. However, these guidelines focus on elective non-cardiac surgery. There are no guidelines for the treatment of proximal femoral fractures, which are considered as semi-urgent surgeries, on dual APT. There is a considerable variability in managing APT patients with proximal femoral fractures between different hospitals. Nevertheless, few studies show the possibility of early surgery in patients on APT. If clopidogrel is interrupted, the ESC-Guidelines recommend a minimal discontinuation of five days prior to surgery [15]. Due to their high age and multiple comorbidities, these patients are especially vulnerable and constitute a demanding challenge for the medical team. For decision making whether the APT should be interrupted and the surgery postponed, or early surgery can be performed with ongoing APT, a clear indication setting and a full medical evaluation are crucial. In patients with a high risk for cardiovascular complications, continuation of APT should be considered. Patients, who take APT as primary prevention for cardiovascular diseases, discontinuation of the APT is a feasible option [11]. 

To analyse the impact of ASS interruption on blood loss, Burger et al. performed a meta-analysis in almost 50 thousand patients. Perioperative on-going ASS increased the risk for bleeding by the factor of 1.5, but it did not increase the level of severity of the bleeding complications. However, withdrawing ASS led to a three times higher risk of cardiovascular events [16]. In the present study, the total blood loss of patients on ASS was not significantly higher compared to patients without APT.

Chechick et al. found a higher blood loss in patients with proximal femoral fractures treated with clopidogrel or dual APT compared to patients without APT [17]. In contrast, Manning et al. did not find an enhanced blood loss in patients on ASS [18]. Results of the present study suggest that early surgery despite of APT may lead to an increased TBL, without increasing the need for RBCs or revision surgery. We found a lower TBL if surgery was delayed for 72 h after APT interruption but no difference in TBL in patients with ongoing APT, regardless of surgical timing. The decrease in TBL after APT interruption may be explained by a partly restored platelet function of the new platelets.

The meta-analysis of Simunovic et al. analysed in 2010 five studies of 4208 patients and showed that an early surgery led to a lower mortality [19]. These findings were conformed in the present study. The randomized pilot study “Hip-Attack” compared patients with early surgery (mean of 6.0 h from admission to surgery) with common timing (mean 24 h). In patients with early surgery, complications occurred in 30%, compared to 47% in patients with common timing [12]. Furthermore, an early surgical intervention may shorten the time of immobility and may lead to a better outcome and lower costs [12]. Additionally, the study of Ekström et al. in 2015 concluded, that a delayed surgery increases the risk for cardiac complications [20]. In the present study, patients who underwent early surgery developed fewer complications during their hospital stay and had a lower mortality. 

Interrupting the APT increases the risk of severe cardiovascular events, such as stent-thrombosis, myocardial infarctions and strokes [21]. Di Minno et al. came to the conclusion, that interrupting clopidogrel within one year after PCI increases the risk for cardiovascular events five to ten times [22]. A perioperative continuation of APT protects patients of cardiovascular complications, but is associated with increased perioperative blood loss [11,14]. Evaluating the bleeding risk versus the risk for thromboembolic complications should be performed individually for each patient [11,23]. In the present study, thromboembolic complications occurred in 1.6% with no difference between patients on APT and the control group without APT. 

The meta-analysis of Soo et al. in 2016 came to the result, that early surgery in patients with proximal femoral fractures and on-going APT can be performed without increased blood loss [24]. The meta-analysis of Siller-Matula et al. showed that preoperative clopidogrel therapy doubled the risk for revision surgery due to major bleeding, without reducing the risk of major adverse cardiac events or mortality in patients who underwent cardiac and non-cardiac surgeries [25]. In contrast, the present study showed that early osteosynthetic surgery despite APT in patients with proximal femoral fractures led to a shorter hospitalization time and a shorter time to surgery. Early surgery despite of APT led to a lower 30-day mortality, less frequent systemic complications as well as less surgical site complications. In addition, on-going APT and early surgery did not cause increased blood loss. Delayed surgery is associated with higher mortality and morbidity and is a major burden for patients due to pain and immobilization. Our findings show that early surgery can be performed safely despite of APT, which leads to a significant increase in patient care.

### Limitations of the Study

The first limitation of the present study is its retrospective design. The second limitation is the inhomogeneous patient population regarding comorbidities, which could have led to prolonged hospital stay, surgical and non-surgical complications and different mortality rates. The third limitation is the relation between the time to surgery and complication rates. Many studies conclude that a prolonged time to surgery leads to a higher complication rate, but a longer time to surgery can also result out of the comorbidities, which are accountable for the occurrence of complications. Nevertheless, we adjusted for these confounders, which should minimize the risk of bias. 

## 5. Conclusions

Early osteosynthetic surgery of proximal femoral fractures despite of APT shortened the time to surgery, the hospitalization length and was associated with less common systemic and surgical site complications as well as a lower mortality and should be considered in eligible patients. 

## Figures and Tables

**Table 1:** Patient demographics regarding antiplatelet therapy (APT). ASA: physical status classification system of the American Society of Anesthesiologists, ASS: acetylsalicylic acid.

	Total	%	Male	%	Female	%	Mean age	ASA
**Total**	396	100	93	23.5	303	76.5	80.4	1.9
**APT**	221	55.8	42	45.2	179	59.1	83.7	1.8
**ASS**	189	47.7	34	36.6	155	51.2	83.6	1.6
**Clopidogrel**	20	5.1	3	3.2	17	5.6	85	2.6
**Dual APT**	12	3	5	5.4	7	2.3	82.1	2.7
**No APT**	174	43.9	51	54.8	124	40.9	76.4	2.1

**Table 2:** Patient demographics including fracture type, type of surgery and comorbidities; DHS: dynamic hip screw, DVT: deep vein thrombosis, PE: pulmonary embolism.

Fracture type	total (%)	APT (%)	no APT (%)	*p*
All fractures	396 (100)	221 (56)	175 (44)	0.024
Femoral neck fracture	81 (20)	41 (51)	40 (49)	1.000
Trochanteric fracture	295 (75)	169 (57)	126 (43)	0.014
Subtrochanteric fracture	20 (5)	11 (55)	9 (45)	0.824
**Type of surgery**				
Screws	29 (7.3)	17 (59)	12 (41)	0.458
DHS	53 (13.4)	24 (45)	29 (55)	0.583
Intramedullary nail	314 (79.3)	180 (57)	134 (43)	0.011
**Comorbidities**				
Arterial hypertension	138 (35)	76 (55)	62 (45)	0.829
Coronary heart disease	46 (12)	40 (87)	6 (13)	0.000
Myocardial insufficiency	13 (3)	6 (46)	7 (54)	0.476
Peripheral artery disease	19 (5)	17 (89)	2 (11)	0.002
Cerebral artery disease or stroke	24 (6)	5 (20)	20 (80)	0.017
Atrial fibrillation	26 (7)	14 (54)	12 (46)	0.835
Diabetes	45 (11)	28 (62)	17 (38)	0.357
History of carcinoma	20 (5)	8 (40)	12 (60)	0.144
Renal insufficiency	20 (5)	9 (45)	11 (55)	0.318
History of DVT or PE	6 (2)	0 (0)	6 (100)	0.006
